# End-of-life treatment preference discussions between older people and their physician before and during the COVID-19 pandemic: cross sectional and longitudinal analyses from the Longitudinal Aging Study Amsterdam

**DOI:** 10.1186/s12877-023-04140-5

**Published:** 2023-07-18

**Authors:** Roosmarijne M. K. Kox, H. Roeline W. Pasman, Annicka G. M. van der Plas, Martijn Huisman, Emiel O. Hoogendijk, Bregje D. Onwuteaka-Philipsen

**Affiliations:** 1 Department of Public and Occupational Health, Amsterdam Public Health Research Institute, Expertise Center for Palliative Care, Amsterdam UMC - Location VU University Medical Center, Amsterdam, The Netherlands; 2 Department of Epidemiology and Data Science, Amsterdam Public Health Research Institute, Amsterdam UMC - Location VU University Medical Center, Amsterdam, The Netherlands; 3 Department of Sociology, Vrije Universiteit Amsterdam, Amsterdam, The Netherlands

**Keywords:** COVID-19, End of life care, Ethics, Communication, Quality of life

## Abstract

**Background:**

COVID-19 could lead to hospitalisation and ICU admission, especially in older adults. Therefore, during the pandemic, it became more important to discuss wishes and preferences, such as older peoples’ desire for intensive treatment in a hospital in acute situations, or not. This study explores what percentage of Dutch older people aged 75 and over discussed Advance Care Planning (ACP) topics with a physician during the first months of the COVID-19 pandemic and whether this was different in these people before the COVID-19 pandemic.

**Methods:**

Data of two ancillary data collections of the Longitudinal Aging Study Amsterdam were used: the LASA 75 PLUS study and the LASA COVID-19 study. The latter provided cross sectional data (during COVID-19; *n* = 428) and longitudinal data came from participants in both studies (before and during COVID-19; *n* = 219).

**Results:**

Most older adults had thought about ACP topics during COVID-19 (76,4%), and a minority had also discussed ACP topics with a physician (20.3%). Thinking about ACP topics increased during COVID-19 compared to before COVID-19 in a sample with measurements on both timeframes (82,5% vs 68,0%). Not thinking about ACP topics decreased in the first months of the COVID-pandemic compared to before COVID-19 for all ACP topics together (68.0% vs 82.2%) and each topic separately (hospital 42.0% vs 63.9%; nursing home 36.5% vs 53.3%; treatment options 47.0% vs 62.1%; resuscitation 53.0% vs 70.7%).

**Conclusions:**

Older people do think about ACP topics, which is an important first step in ACP, and this has increased during COVID-19. However, discussing ACP topics with a physician is still not that common. General practitioners could therefore take the initiative in broaching the subject of ACP. This can for instance be done by organizing information meetings.

**Supplementary Information:**

The online version contains supplementary material available at 10.1186/s12877-023-04140-5.

## Background

Early 2020, the world was confronted by the acute threat of the corona virus disease 2019 (COVID-19), of which the course was unpredictable. In the Netherlands, it has infected more than 1.7 million people between February 2020 and July 2021 [1]. Older people seem to be at higher risk for getting seriously ill or die due to COVID-19; in the years 2020 – 2022 93–95% of all COVID-19 deaths were among Dutch people aged 65 years and above [1, 2]. In addition, more than 65 thousand people were hospitalized due to a COVID-19 infection until July 2021, mostly older people, of which part needed to be admitted to an Intensive Care Unit, at which ventilatory support could be needed [1, 3, 4].

COVID-19 quickly became a much discussed topic in Dutch media, among which the discussion about who should and should not be admitted to an Intensive Care Unit (ICU) when hospital beds would become too scarce (also known as ‘code black’) [5]; a discussion that mainly focused on potential consequences for older people. Alongside this discussion, for older adults the importance of thinking about and discussing treatment wishes may have become more clear, especially the importance of talking about goals and preferences with their general practitioner. Moreover, Dutch general practitioners got encouraged by the Dutch College of General Practitioners to discuss treatment wishes with their older patients, because of higher risks among these patients in getting severely ill due to COVID-19 [6]. To help people to define personal goals and preferences for future medical treatment and care, talk about it with relatives and healthcare professionals and record and review these goals and preferences, Advance Care Planning (ACP) can help [7]. Important topics to discuss include end-of-life treatment preferences such as place of care, treatment options, and hospitalization [8]. A former Dutch study found that 32,6% of adults 57 years and older had had ACP discussions with a physician or had filled in an advance directive [9]. ACP is found to improve the quality of end of life care and satisfaction, [10, 11] and it results in less in-hospital deaths and an increased use of hospice care [11]. A Dutch guideline states that preferably, ACP is discussed with the general practitioner or physician with whom the patient has a good treatment relationship. Important in ACP is talking with a physician in advance about personal goals and preferences at the end of life, to prevent having to start thinking about it in acute situations [6].

Since COVID-19 could lead to hospitalization, it is important for healthcare professionals to know wishes of their patients, such as whether they desire intensive treatment in a hospital in acute situations, or not [6]. A Dutch qualitative study among nursing home physicians found COVID-19 specific changes in ACP, such as a COVID-19 infection as a reason for initiating ACP and intensive care unit admission as an additional topic during ACP conversations [12]. Furthermore, the serious threat of COVID-19, the media attention including the discussions on ‘code black’ and the encouragement of general practitioners to discuss treatment wishes with their older patients might have caused an increase in ACP in older adults. Therefore, this study explores what percentage of Dutch older people aged 75 and over discussed ACP topics with a physician during the first months of the COVID-19 pandemic and whether this was different in these people before the COVID-19 pandemic. We investigated three research questions: (1) What percentage of older adults discussed ACP topics with a physician during the first months of the COVID-19 pandemic? (2) What are characteristics of older adults discussing ACP with a physician during the first months of the COVID-19 pandemic, regarding one or more of four ACP topics and specifically the topic of hospitalization? (3) And does the prevalence of discussing ACP topics with a physician differ between before and during the COVID-19 pandemic?

## Methods

### Design

We used data of two ancillary data collections of the Longitudinal Aging Study Amsterdam (LASA): the LASA 75 PLUS study and the LASA COVID-19 study [13, 14]. LASA is an ongoing longitudinal cohort study on the physical, emotional, cognitive, and social functioning of a nationally representative sample of middle-aged and older adults in the Netherlands. Sampling and data collection of the LASA study have been described in detail elsewhere [13].

The LASA 75 PLUS study was performed between 2016 and 2019 among LASA participants who were aged 75 years and older at the time (all were born before 1941), which resulted in three nine-monthly measurements (wave I-v1, wave I-v2 and wave I-v3). Of *N* = 686 invited LASA participants, *N* = 601 persons agreed to participate (87.6%). In our study we used data from the third wave (I-v3). It was sent out to 550 persons (all respondents of the second wave), of which 507 participated (92.2%). Data collection was done by a face-to-face home interview or, if that was not possible, by a short telephone interview. Sampling and data collection of the LASA 75 PLUS study have been described in detail elsewhere [13]. Below is a detailed description of the sample used in this paper.

The LASA COVID-19 study was added in between the LASA measurement cycle of 2018–2019 and the planned measurement cycle of 2021–2022, in order to capture the impact of the COVID-19 pandemic on the daily lives of older adults. The LASA COVID-19 questionnaire was sent after the first COVID-19 wave in the Netherlands, on June 8, 2020. Of *N* = 1,701 LASA participants who were participating in the last LASA measurement cycle (Wave J, 2018–2019), *N* = 1,485 participants were invited to participate. Participants not invited (*n* = 216) either already had died (*n* = 61) or were purposely not selected (*n* = 155) due to an expected high burden in filling in the questionnaire (respondents who already had a short telephone interview or proxy interview in the 2018–2019 measurement cycle). These 155 persons were on average older (82.3 years, SD 9.0) than those 1,485 invited to participate (73.4 years, SD 7.6). The questionnaire could be completed in writing, digitally or in exceptional cases in a telephone interview. Data of *N* = 1,128 participants (76%) aged 62 to 102 years were recorded between June 9, 2020 and October 8, 2020. Sampling and data collection of the LASA COVID 19 study have been described in more detail elsewhere [14]. Below is a detailed description of the sample used in this paper.

### Sample

**Fig. 1:**
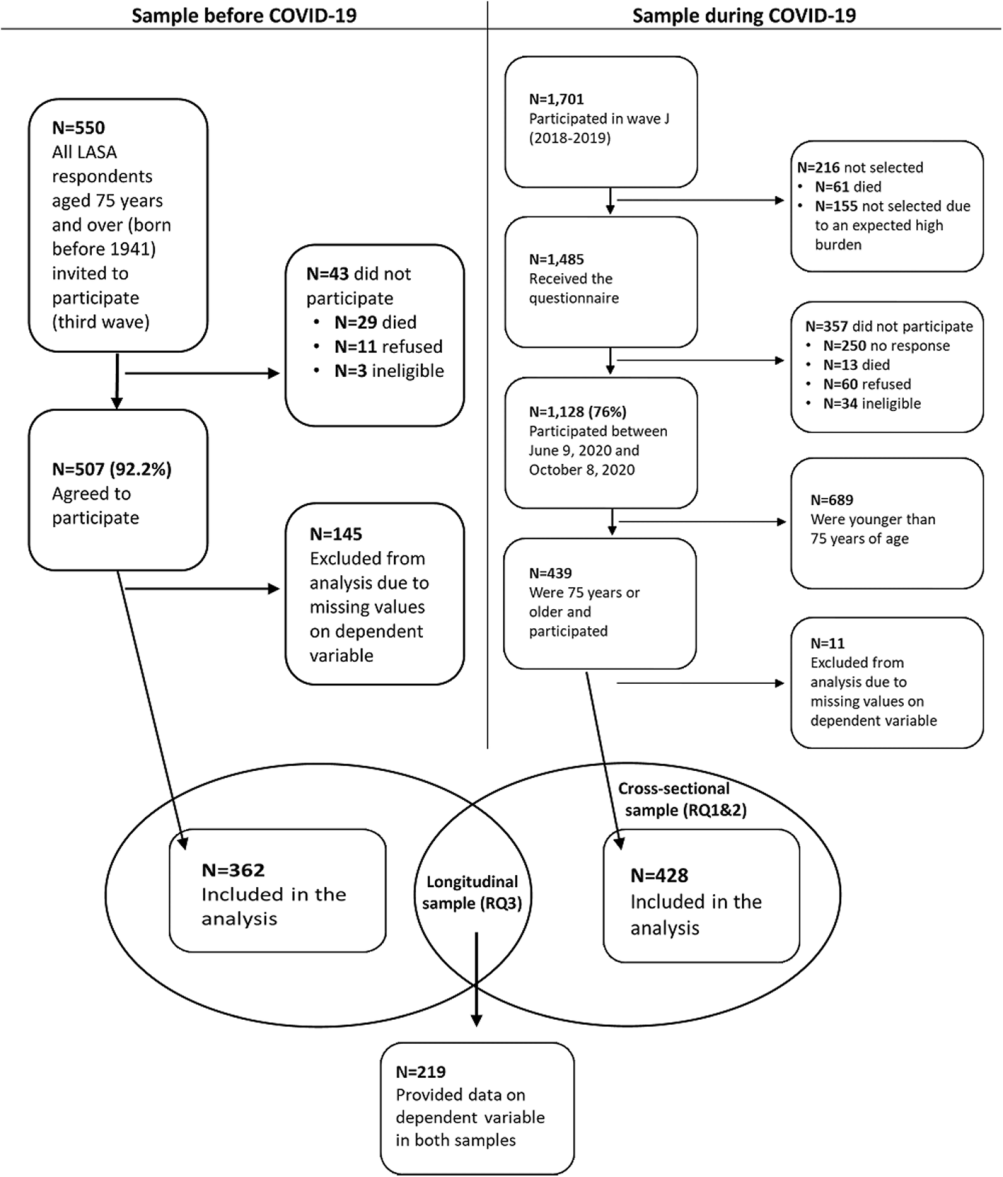
Selection of study samples

This study used data of wave I-v3 (January 2018-January 2019) of the LASA 75 PLUS study and data from the LASA COVID-19 study. Figure 1 shows the selection of both study samples. The cross-sectional part of this study (RQ 1 and 2) consisted of *N* = 439 people aged 75 years and older who completed the LASA COVID-19 questionnaire, of which *N* = 11 participants were excluded from the analysis because of missing values on all dependent variables. The pre-COVID sample consisted of *N* = 507 people aged 75 years and older who completed the third wave (I-v3), of which *N* = 145 were excluded from the analysis because of missing values on the dependent variables (143 of which were administered the short telephone interview that did not include the questions on ACP behaviour). The longitudinal sample (RQ 3) consisted of 219 people aged 75 years and older who had data on both the LASA 75 PLUS Study (wave I-v3) and the LASA COVID-19 study.

### Measurement instruments

#### Dependent variables

In both ancillary studies (LASA 75 PLUS study and LASA COVID-19 study), participants were asked whether they had thought about the following Advance Care Planning (ACP) topics regarding the end of life and whether they discussed ACP topics with a physician during the past months (yes/no): (1) willing to go to a hospital in certain circumstances, (2) willing to be admitted to a nursing home, (3) which treatment options you would be willing to undergo in certain circumstances, (4) and wanting to be resuscitated. For each of the four ACP topics a dependent variable was created in which the two questions were combined, resulting in three possible answer categories: (1) did not think about it; (2) thought about it, but did not discuss it with a physician; (3) thought about it and discussed it with a physician. Another dependent variable with the same three categories was made, by combining all four ACP topics in order to look at ACP in general, in which they had thought or discussed one or more of the four ACP topics.

#### Independent variables

We examined the following characteristics of participants: age, sex, partner status, education level, housing, depressive symptoms, anxiety symptoms, mastery, number of activities with some difficulty or worse, self-perceived health, loneliness, having been in quarantine, having been ill during the COVID-19 crisis, knowing someone who tested positive on COVID-19, knowing someone who was hospitalized for COVID-19, knowing someone who died from COVID-19 and discussed ACP with someone other than a physician. Details on the COVID related variables can be found in [14], and information on all other variables are on the LASA website [15].

### Analysis

Descriptive statistics were used to describe the study population and the prevalence of discussing ACP topics with a physician during the first months of the COVID-19 pandemic (RQ1). Univariate logistic regression analyses were performed to identify characteristics of older adults discussing ACP topics with a physician during COVID-19 (RQ2), adjusted for age and sex. This analysis was done for the variable combining the four ACP topics. In addition, we also did this analysis for ‘willing to go to a hospital in certain circumstances’ specifically, because this topic was especially important during the COVID-19 situation. The two dependent variables were dichotomized into ‘did not discuss it with physician’ (categories 1 and 2 combined) and ‘did discuss it with physician’ (category 3). Finally, to compare the prevalence of discussing ACP topics with a physician before and during the COVID-19 pandemic (RQ3), descriptive statistics were used and confidence intervals were calculated. IBM SPSS version 26 (IBM Analytics) was used to carry out the statistical analyses.

## Results

### Study population

Characteristics of the cross-sectional sample (RQ 1 and 2) and longitudinal sample (RQ 3) are presented in Table 1. In both samples about three quarter of the participants were aged between 75 and 84 years (76.9% and 70.3%), little over half was female (53.5% and 58.4%) and had a partner (60.0% and 50.2%), about three quarter had a low/middle level of education (75% and 75.3%), and almost everyone was living independently (98.5% and 98.1%). Housing was not included in further analyses, because of a low number of participants who lived dependently (*n* = 6).

**Table 1:** Characteristics of the study population according to the two samples (absolute numbers and rounded %)

	**Cross-sectional sample** ^a^ **(** ***n*** ** = 428)**		**Longitudinal sample** ^b^ **(** ***n*** ** = 219)**	
	**n**	**%**	**n**	**%**
**Age**				
***75—79***	190	44.4	48	21.9
***80—84***	139	32.5	106	48.4
***85—89***	73	17.1	50	22.8
***90*** ** + **	26	6.1	15	6.8
**Sex**				
***Male***	199	46.5	91	41.6
***Female***	229	53.5	128	58.4
**Partner status**				
***Partner***	257	60.0	110	50.2
***No partner***	171	40.0	109	49.8
**Education level**				
***Low***	161	37.6	90	41.1
***Middle***	160	37.4	75	34.2
***High***	107	25.0	54	24.7
**Housing**				
***Independent***	391	98.5	212	98.1
***Dependent***	6	1.5	4	1.9

^a^Response between June 9, 2020 and October 8, 2020

^b^Characteristics of fixed variables were measured at baseline (at inclusion in wave J). Age was reported as a mean age between the covid measurement and the third wave of the 75 PLUS study

### Prevalence of thinking about and discussing ACP topics with a physician, during COVID-19

**Fig. 2:**
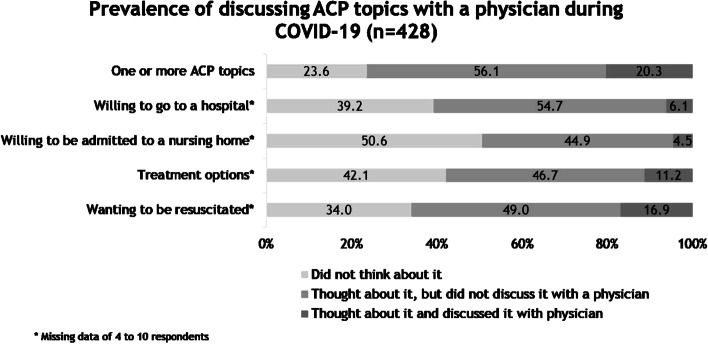
Prevalence of discussing ACP topics with a physician during COVID-19 (*n* = 428)

Figure 2 shows that most (76.4%) participants had thought about one or more of the ACP topics, but did not discuss them with a physician (56.1%) and 20.3% had thought and also discussed this with a physician. During COVID-19, most participants considered whether they were willing to go to a hospital in certain circumstances, which treatment options they would (not) want in certain circumstances or whether they would want to be resuscitated, but did not discuss these topics with a physician (resp. 54.7%, 46.7%, and 49.0%). Moreover, about half of the participants did not think about whether they were willing to be admitted to a nursing home (50.6%). Of the four specific ACP topics, resuscitation was most often discussed with a physician (16.9%).

### Characteristics of persons discussing one or more ACP topics with a physician, during COVID-19

In comparison to those who did not discuss any of the ACP topics with their physician, participants who discussed at least one of them with their physician more often lived in a residential home (compared to home) (OR 6.57 [1.07–40.47]), had more often two or more activities with some difficulty or worse (OR 3.14 [1.54–6.39]), had more often discussed ACP topics with someone other than a physician (OR 9.10 [4.54–18.23]) and more often did not know someone who died from COVID-19 (OR 2.01 [1.02–3.94]) (see Table 2). In Additional File 1 we report on the ACP variables in more detail (providing percentages regarding not thought about it, thought about it, discussed it with a physician).

**Table 2:** Logistic regression analyses of discussing ACP topics with a physician during COVID-19, adjusted for age and sex (*n* = 428)

	**Discussing one or more of the ACP topics with physician**		**Discussing hospitalization with physician**	
	**Discussed it with physician** **(** ***n*** ** = 87)** **n (%)**	Adjusted OR* **(95% CI)**	**Discussed it with physician** **(** ***n*** ** = 26)** **n (%)**	Adjusted OR* **(95% CI)**
Education level				
*Low*	32 (36.8)	Ref	11 (42.3)	Ref
*Middle*	32 (36.8)	1.16 (0.65–2.07)	6 (23.1)	0.59 (0.21–1.65)
*High*	23 (26.4)	1.52 (0.80–2.92)	9 (34.6)	1.23 (0.47–3.25)
Not having a partner	46 (52.9)	1.29 (0.74–2.23)	11 (42.3)	1.01 (0.40–2.55)
Living in residential home (vs home)^b^	4 (5.1)	6.57 (1.07–40.47)	2 (8.0)	6.02 (0.92–39.14)
Depressive symptoms	6 (6.9)	2.55 (0.82–7.88)	3 (11.5)	4.22 (1.03–17.26)
Anxiety symptoms^a^	13 (14.9)	1.37 (0.67–2.79)	6 (23.1)	2.61 (0.97–7.03)
Mastery^a^ [mean (SD)]	23.53 (4.94)	0.96 (0.91–1.02)	23.36 (5.71)	0.95 (0.87–1.04)
Number of activities with some difficulty or worse (out of 7)^b^				
*0 activities*	13 (15.7)	Ref	3 (11.5)	Ref
*1 activity*	19 (22.9)	2.12 (0.98–4.62)	9 (34.6)	4.05 (1.05–15.65)
≥ *2 activities*	51 (61.4)	3.14 (1.54–6.39)	14 (53.8)	3.40 (0.89–12.95)
Less than good self-perceived health (vs good/excellent)^b^	27 (35.5)	1.07 (0.61–1.87)	11 (52.4)	2.39 (0.96–5.96)
Loneliness, no^a^	41 (47.7)	1.49 (0.90–2.46)	12 (46.2)	1.19 (0.53–2.68)
Having been in quarantine^b^	12 (14.3)	1.06 (0.52–2.19)	5 (20.0)	1.88 (0.66–5.40)
Not having been ill during the COVID-19 crisis^a^	74 (89.2)	1.29 (0.58–2.84)	22 (84.6)	0.71 (0.23–2.22)
Knowing someone who tested positive on COVID-19**				
*Mentioned*	24 (27.6)	Ref	6 (23.1)	Ref
*Not mentioned*	63 (72.4)	0.96 (0.56–1.62)	20 (76.9)	1.19 (0.46–3.06)
Knowing someone who was hospitalized for COVID-19**				
*Mentioned*	13 (14.9)	Ref	4 (15.4)	Ref
*Not mentioned*	74 (85.1)	1.52 (0.79–2.95)	22 (84.6)	1.51 (0.50–4.60)
Knowing someone who died from COVID-19**				
*Mentioned*	12 (13.8)	Ref	3 (11.5)	Ref
*Not mentioned*	75 (86.2)	2.01 (1.02–3.94)	23 (88.5)	2.31 (0.67–7.95)
Discussed ACP with someone other than a physician^b^	71 (86.6)	9.10 (4.54–18.23)	16 (80.0)	8.18 (2.62–25.55)

*n* number of participants, *SD* standard deviation, *CI* confidence interval, *Ref* reference

^*^Adjusted for age and sex

^**^Knows someone: partner, parent, child, sibling, grandchild, other family, neighbour, friend/acquaintance, someone else

^a^Less than 5% missing values

^b^Above 5% missing values; housing: 7.2%, number of activities with some difficulty or worse (out of 7): 7.7% / 7.1%, self-perceived health: 10.7%, having been in quarantine: 5.6% / 5.4% and talked to someone else: 7.7% / 13.4%

### Characteristics of persons discussing hospitalization with a physician, during COVID-19

In comparison to those who did not discuss hospitalization with their physician, participants who discussed hospitalization with their physician had more often depressive symptoms (OR 4.22 [1.03–17.26]), had more often one activity with some difficulty or worse (OR 4.05 [1.05–15.65]) and had more often discussed ACP topics with someone other than a physician (OR 8.18 [2.62–25.55]) (see Table 2).

### Differences in prevalence of thinking about and discussing ACP topics with a physician before and during COVID-19

Table 3 shows that *not thinking* about ACP topics statistically significant decreased in the first months of the COVID-pandemic compared to before COVID-19 for all ACP topics together and each topic separately. *Discussing* ACP topics with a physician increased in the first months of the COVID-pandemic compared to before COVID-19 for all ACP topics, but not statistically significant.

**Table 3:** Prevalence of discussing ACP topics with a physician in the past months, before and during COVID-19 (*n* = 219)

	**Before COVID-19**			**During COVID-19**		
	**n**	**%**	**CI**	**n**	**%**	**CI**
**One or more ACP topics**						
*Did not think about it*	70	32.0	26.1–38.3	39	17.8	13.2–23.3
*Thought about it, but did not discuss it with physician*	101	46.1	39.6–52.7	123	56.2	49.5–62.6
*Thought about it and discussed it with physician*	48	21.9	16.8–27.7	57	26.0	20.6–32.1
**Willing to go to a hospital**						
*Did not think about it*	127	58.0	51.4–64.4	79	36.1	29.9–42.6
*Thought about it, but did not discuss it with physician*	73	33.3	27.3–39.8	121	55.3	48.6–61.7
*Thought about it and discussed it with physician*	19	8.7	5.5–12.9	19	8.7	5.5–12.9
**Willing to be admitted to a nursing home**						
*Did not think about it*	139	63.5	56.9–69.6	100	46.7	40.1–53.4
*Thought about it, but did not discuss it with physician*	73	33.3	27.3–39.8	100	46.7	40.1–53.4
*Thought about it and discussed it with physician*	7	3.2	1.4–6.2	14	6.5	3.8–10.4
**Treatment options**						
*Did not think about it*	116	53.0	46.4–59.5	81	37.9	31.6–44.5
*Thought about it, but did not discuss it with physician*	76	34.7	28.6–41.2	102	47.7	41.0–54.3
*Thought about it and discussed it with physician*	27	12.3	8.5–17.2	31	14.5	10.3–19.7
**Wanting to be resuscitated**						
*Did not think about it*	103	47.0	40.5–53.6	63	29.3	23.5–35.6
*Thought about it, but did not discuss it with physician*	77	35.2	29.1–41.6	107	49.8	43.1–56.4
*Thought about it and discussed it with physician*	39	17.8	13.2–23.3	45	20.9	15.9–26.7

*n* number of participants, *CI* confidence interval

## Discussion

We found that more than half of the Dutch participants aged 75 years and older had thought about one or more of four ACP topics during the first months of the COVID-19 pandemic and about their willingness to be hospitalized specifically (resp. 76.4%; 60.8%), but only a minority had also discussed it with a physician (resp. 20.3%; 6.1%). People who discussed one or more of the ACP topics with a physician during COVID-19 were living on average more often dependently in a residential home. Furthermore, they had on average more often two or more activities with some difficulty or worse, had discussed ACP with someone other than a physician and did not know someone who died from COVID-19. Discussing willingness to be hospitalized with a physician during COVID-19 was significantly associated with having depressive symptoms, having one activity with some difficulty or worse, and discussing ACP with someone other than a physician. Our results show that during the first months of the COVID-19 pandemic thinking about ACP topics has increased in comparison to before COVID-19. Furthermore, discussing ACP topics with a physician has slightly, yet not significantly increased during COVID-19 compared to before COVID-19.

### Prevalence of discussing ACP topics with a physician during COVID-19

In our study, most older adults had thought about ACP topics during COVID-19, but had not discussed them with a physician. Two other studies among Dutch older adults aged 75 years and above (before COVID-19), using different Dutch data, found similar prevalence regarding ACP [16, 17]. These two studies also found that most older adults think about ACP topics, however that discussing ACP with a physician is still not that common [16, 17]. However, also discussing your preferences and wishes with a physician is found to be important for receiving end-of-life care in accordance with preferences and preventing unnecessary and undesirable hospital admissions [18]. General practitioners are in a good position to have ACP conversations because of their long-term relationship [19]. Patients think it is important that their general practitioner initiates those discussions [20].

### Difference in discussing ACP topics with a physician during COVID-19 compared to before

We found an increase in thinking about ACP topics during COVID-19 compared to before COVID-19. This increase might be due to the constant threat of COVID-19, the attention in media and the attention among general practitioners for the relevance of ACP [6]. While it would be expected that ACP topics would have been discussed more frequently with a physician due to the attention in media and the imposed attention among general practitioners by the Dutch College of General Practitioners [6], our study shows that it has not necessarily led to an increase in discussing ACP topics with a physician. This might be due to earlier found barriers of adults aged 50 and above to talk to their physician about ACP topics: feeling too young and too healthy for ACP, ACP is a too emotional topic, ACP is the responsibility of the physician, there is not enough time in appointments, physicians are too busy, lack of trust in physicians, lack of knowledge about ACP, and too many other medical problems to talk to a physician about [21, 22]. During COVID-19, some of these barriers might have been especially important, such as the perception of patients that the general practitioners were too busy. Although physicians were stimulated by the Dutch College of General Practitioners to contact older patients by phone, both older patients and physicians may prefer to have ACP conversations in person. In addition, recent studies in the United States and the Netherlands show that between 20–41% of older adults avoided care during the COVID-19 pandemic, and avoidance of care was related to older age [23–25]. Furthermore, a Dutch study found that another reason for patients to not talk to a physician about ACP could be that their loved ones knew what they wanted, therefore it is needless [16]. Our study, however, shows that older adults more often discussed ACP topics with a physician if they also had discussed ACP topics with someone other than a physician. Nevertheless, we do not know whether respondents did not discuss ACP topics with a physician due to not being ready for it, because they felt there was no opportunity for it due to the work pressure among physicians during COVID-19, or another reason.

### Stimulating ACP discussions with physicians

Looking at the Stages of Change Model [26] and a conceptual model of ACP [27], it seems like the majority of older adults in our study were in the pre-contemplation phase before COVID-19, because most older adults had not thought about ACP topics. During COVID-19, a number of older adults may have shifted to the contemplation phase, as a majority of participants had thought about ACP topics but did not discuss it with a physician. As we found that older people who discussed ACP with their family or friends were more likely to discuss ACP with their physician, people who discussed ACP with their family or friends seem to be in the next phase of change: the preparation phase. This association was also found in an earlier study in the United States among adults aged 50 and above [27].

One way to stimulate ACP discussion with physicians therefore seems to be stimulating people to think and discuss their wishes and preferences with family or friends. Information meetings about ACP given by the general practitioner might be a good way to do this. We found earlier that such meetings stimulate ACP discussion with physicians, but even more with others, like family or friends [17].

### Strengths and limitations

Major strengths of this study are the broad range of measurements available within LASA, the fact that the COVID-19 questionnaire was distributed immediately after the first COVID-19 wave, leaving little recall bias, and the longitudinal design of this study, which allowed us to compare discussing ACP topics with a physician before COVID-19 with the first months of the COVID-19 pandemic. However, this study also has limitations. Our study population was relatively small, making further analyses impossible. Another limitation of this study is that we lack details on why older adults did not discuss ACP topics with their physician. In our longitudinal subsample, the percentage of people who did not think about ACP topics during COVID-19 was lower (17.8%), compared to the larger (*n* = 428) COVID sample (23.6%). This could be a result of the case mix in the sub selection, since the group of 428 persons appears to be younger. Furthermore, the COVID-19 study only has data available from participants who were healthy enough to fill out the questionnaire. Therefore, some groups are underrepresented in the sample, such as the oldest old and those who are more frail.

## Conclusion

Older people do think about ACP topics, which is an important first step in ACP, and this has increased during COVID-19. However, discussing ACP topics with a physician is still not common. General practitioners could therefore take the initiative in broaching the subject of ACP. This can for instance be done by organizing information meetings.

## Supplementary Information

**Additional file 1.**

## Data Availability

Access to data from the Longitudinal Aging Study Amsterdam can be requested by submitting a LASA analysis proposal form for evaluation. The form is available on this website: https://lasa-vu.nl/en/request-data/. The LASA evaluation committee provides access to the data on the condition that the goals of the data request are in keeping with the overarching aims of LASA that its participants have provided consent for. The LASA analysis proposal template includes the option to request data for replication purposes. Further questions can be e-mailed to lasa@amsterdamumc.nl.
