# Supplementary material for: End-of-life treatment preference discussions between older people and their physician before and during the COVID-19 pandemic: cross sectional and longitudinal analyses from the Longitudinal Aging Study Amsterdam
Source: BMC Geriatr. 2023 Jul 18;23:441. doi: 10.1186/s12877-023-04140-5 (PMC10355077; doi:10.1186/s12877-023-04140-5)
Supplement: Supplementary file 1 — Additional file 1. [file 12877_2023_4140_MOESM1_ESM.pdf]

Additional File 1

Table A1. One or more ACP topics; never thought about it, thought about it, discussed with a physician during COVID-19 (n=428) *

	Never thought about it (n=101) n (%)	Thought about it, but never discussed it with physician (n=240) n (%)	Discussed it with physician (n=87) n (%)
Education level			
Low	47 (46.5)	82 (34.2)	32 (36.8)
Middle	34 (33.7)	94 (39.2)	32 (36.81)
High	20 (19.8)	64 (26.7)	23 (26.4)
**Having a partner, no**	**28 (27.7)**	**97 (40.4)**	**46 (52.9)**
**Living in residential home (vs home)**	**0 (0)**	**2 (0.6)**	**4 (5.1)**
**Depressive symptoms**	**0 (0)**	**8 (3.3)**	**6 (6.9)**
**Anxiety symptoms**	**4 (4.0)**	**32 (13.5)**	**13 (14.9)**
**Mastery [mean (SD)]**	**26.11 (4.44)**	**24.48 (4.43)**	**23.53 (4.94)**
**Number of activities with some difficulty or worse (out of 7)**			
0 activities	**46 (48.9)**	**80 (36.7)**	**13 (15.7)**
1 activity	**23 (24.5)**	**52 (23.9)**	**19 (22.9)**
≥2 activities	**25 (26.6)**	**86 (39.4)**	**51 (61.4)**
Less than good/excellent self-perceived health (vs good/excellent)	21 (24.1)	71 (32.4)	27 (35.5)
Loneliness, no	46 (46.9)	91 (38.7)	41 (47.7)
Having been in quarantine	7 (7.4)	33 (14.7)	12 (14.3)
Not having been ill during the COVID-19 crisis	85 (89.5)	201 (86.6)	74 (89.2)
Knowing someone who tested positive on COVID-19, m*entioned*	27 (26.7)	64 (26.7)	24 (27.6)

Knowing someone who was hospitalized for COVID-19, *mentioned*	18 (17.8)	58 (24.2)	13 (14.9)
**Knowing someone who died from COVID-19, m*entioned***	**17 (16.8)**	**66 (27.5)**	**12 (13.8)**
**Discussed ACP with someone other than a physician**	**N.A.**	**125 (59.0)**	**71 (86.6)**

* missing n=11; bold highlights p<0.05 in chi-squared test or Anova (mastery); SD = standard deviation; N.A. = not applicable.
